# Does It Take a Village? The Impact of LGBTQ+ Community and Geographic Location on Associations among Parenting Stress, Parent Mental Health, and Child Adjustment

**DOI:** 10.3390/ijerph21091206

**Published:** 2024-09-12

**Authors:** Kevin A. McAweeney, Rachel H. Farr

**Affiliations:** Department of Psychology, University of Kentucky, Lexington, KY 40506, USA

**Keywords:** LGBTQ+, family, adolescents, sense of community, mental health, child adjustment

## Abstract

While LGBTQ+-parented families share many similarities with their cis-het parent counterparts, they still experience unique factors exclusive to them. One such factor is access to the LGBTQ+ community. Utilizing a diverse sample of LGBTQ+ parents with adolescents in the U.S., primarily living in Southern and Midwest states, we examined the potential moderating impact of a parent’s sense of LGBTQ+ community on the relationship between parenting stress, parent mental health, and child adjustment. Regression analyses demonstrated a series of positive associations between LGBTQ+ parent stress, parent mental health concerns, and child adjustment issues. However, sense of community failed to moderate these associations. Parent sexual identity, age, and recruitment method were found to have unique associations with outcome variables. Implications for policy, clinical practice, and future research are discussed.

## 1. Introduction

Overall, LGBTQ+-parented families illustrate minor differences in both child development and adjustment, as well as parent–child relationships, when compared to cisgender heterosexual (cis-het)-parented families [1]. While LGBTQ+-parented families in the U.S. have seen major expansion in their rights thanks to Obergefell v. Hodges leading to marriage equality for same-sex couples, they are not treated the same, socially or legally, as cis-het-parented families [2,3,4,5,6,7]. Thus, strengths-based research is needed that illustrates how LGBTQ+-parented families are, in fact, well-adjusted and healthy to dispel negative stereotypes that align with negative treatment [8]. This research should emphasize the unique benefits that LGBTQ+ parents bring to the family system. There is also presently a lack of LGBTQ+ research outside of coastal regions in the U.S. [9] and LGBTQ+ family research that investigates LGBTQ+ families with adolescent children.

One such unique aspect of having an LGBTQ+ parent is the potential for that parent to gain support via the LGBTQ+ community. This community connection may also have a unique role with adolescent children of LGBTQ+ parents, in part due to the importance of identity during adolescence [10] and the distinct role that children of LGBTQ+ parents represent within the broader LGBTQ+ community [11]. Using Ecological Systems Theory [12], it would stand to reason that an LGBTQ+ parent’s sense of community would impact the child. Within this theory, parent–child interaction occurs within the microsystem, which is acted upon by outside factors such as LGBTQ+ community support (mesosystem) and broader cultural stigma toward LGBTQ+ individuals or parents (macrosystem).

Prior LGBTQ+ research examining community has found that LGBTQ+ community connectedness can act as a protective factor, moderating the association between perceived stigma and mental health concerns [13]. Community connectedness has also been found to be associated with lower levels of psychological distress and higher levels of social well-being [14]. Notably, sense of LGBTQ+ community and connection to the community are not equal for all LGBTQ+ individuals. Those in spaces or situations with less LGBTQ+ support may rely on the internet to engage with the LGBTQ+ community [15,16]. Research on LGBTQ+ parents in Australia and New Zealand found that feelings of support from the LGBTQ+ community varied based on living situation, with those living in the inner city reporting more support than other locations [17]. Different LGBTQ+ identities may also experience differing levels of sense of community, such as bisexual individuals demonstrating lower levels of sense of connection to the LGBTQ+ community than gay and lesbian individuals [18]. In that same research, the associations between bisexual identity and affective symptoms and mental well-being were found to be mediated by sense of connection to the LGBTQ+ community [18]. Together, this research highlights the important protective role the LGBTQ+ community can play in the role of LGBTQ+ individuals while also highlighting the complex role identity and living situation can play in this dynamic.

Alongside parent LGBTQ+ community belonging, broader cultural stigma and attitudes (mesosystem) should impact the family system. With varying LGBTQ+-related laws across states, it stands to reason that LGBTQ+ individuals would face unique issues across geographic regions. However, LGBTQ+ research outside of coastal regions in the U.S. is limited [9], in spite of the fact that geographic region plays a unique role in the lives of LGBTQ+ individuals. This gap in the research is especially relevant, as the Trevor Project notes that LGBTQ+ youth in the South have greater odds of suicide attempts compared to youth in other regions [19], indicating a dire mental health disparity across regions. The Southern LGBTQ Health Survey indicates similar mental health concerns across more general LGBTQ+ Southern U.S. samples [20]. These findings may pertain to the overall political and social climate of the Southern United States. The states within the U.S. South are predominantly conservative and religious [21]. The Religious Right has been historically at odds with the LGBTQ+ community [22], which often has been reflected in laws and public policy that take root in regions such as the U.S. South. The Movement Advancement Project’s [23] Policy Tally, a score based around inclusivity and protective laws within each state, reflects this, with 93% of LGBTQ+ individuals living in the South residing in a state with a low or negative tally score. This score takes into account a variety of topics such as parental recognition, criminal justice, nondiscrimination policies, and health care. Only a single state in the South has above a “low” score (Virginia with “fair”). This hostile climate could account for the dire mental health concerns LGBTQ+ individuals in the South face. With this in mind, we believe research that is inclusive of LGBTQ+ individuals from less represented regions in the U.S. is critical, as is research diving deeper into the relationship between U.S. region of residence and the mental well-being of LGBTQ+ people and their families.

While LGBTQ+ families may be uniquely impacted by sense of LGBTQ+ community belonging and geographic region, they should also demonstrate similar interactions between mental health variables as other cis-het-parented families, namely, as suggested by Ecological Systems Theory [12], an interconnection between parent and child mental well-being. Research with LGBTQ+ parents has previously found parent depressive symptoms to predict internalizing and externalizing issues in children [24] and parenting stress to function as a predictor of children’s externalizing problems [25]. In adoptive family research with a sample that includes a large number of LG couples, parent depressive symptoms were linked to increased postplacement parent stress [26]. Together, this illustrates a series of links between parent stress, parent mental health, and child adjustment. Notably, this research tends to focus on LGBTQ+ parents with young children as opposed to those with adolescent children.

### Hypothesis and Research Question

Based on previous research surrounding associations between parenting stress and mental health concerns among parents [27], parenting stress and child adjustment [25,28,29], and parents’ mental health and their children’s adjustment [30,31,32,33,34], as well as research surrounding the benefits of parental belonging to the LGBTQ+ community on children [35], we hypothesize that there will be a set of positive associations between parent mental health (depression and anxiety), child adjustment issues, and parenting stress. We also predict that sense of LGBTQ+ community will have an indirect effect on these relationships, acting as a moderator. Specifically, we predict that it will act as a protective factor, with parents higher in sense of LGBTQ+ community exhibiting better outcomes. In line with Ecological Systems Theory [12] and informed by legal and societal changes faced by LGBTQ+ individuals, we also examine geographic region as a potential covariate. Suspecting that differential experiences with regard to stigma and prejudice may impact family outcomes, leaning upon minority stress theory [36,37], and with the knowledge that prior use of our various measures have shown differences across racial [38,39], sexual [40], and gender identities [41], we examined child and parent race, parent gender, and parent sexual identity as potential covariates. Since this study focuses on the impact of LGBTQ+ community belonging, we also considered adolescent sexual and gender identity (i.e., being LGBTQ+) as a potential covariate. Lastly, with the knowledge that depression and anxiety appear to decline with age [42], parent age was examined as a potential covariate.

## 2. Materials and Methods

The data for this study come from the Queer Parents and Adolescent Lives Study (QPAL). It is a study of LGBTQ+ parents and their adolescent children ages 12-19 years. Families represented multiple parenthood pathways, including adoption, surrogacy, and children from reproductive sex. While families were gathered from across the U.S., special focus was placed on recruiting LGBTQ+ families living within the U.S. South and Midwest to address a lack of LGBTQ+ research representing those regions [9]. Surveys were given via Qualtrics, with data gathered across several psychological scales, along with demographic data of parents and their children. Attention checks were put in place to ensure the quality of responses (e.g., “To make sure you are reading this please write “pasta” in the box below”). Both parents and children were eligible for the project, but the current study is focused on parent data. Participants were compensated USD 35 for participation. Data collection began near the end of 2021 and was finalized in 2024. Data are not currently publicly available but may be attained from authors upon reasonable request.

### 2.1. Participants

The QPAL study consists of 150 parent participants from the United States. Participants were recruited through snowball sampling utilizing email outreach (*n* = 79) and also through Prolific (*n* = 71). Our parent sample tended to skew female (60%) and towards plurisexual identities (55% bisexual/pansexual/omnisexual). A total of 35% of our sample were same-gender-attracted parents (*n* = 52). A single nonbinary participant identified as heterosexual. The average participant age was 40.62 years. In terms of racial/ethnic identity, our data primarily consist of white participants (64% white). Families were primarily from the U.S. South, with a substantial portion coming from the Midwest (i.e., 43% South, 26% Midwest, 16% Northeast, 15% West). For full parent demographics, see Table 1.

Child racial demographics are more diverse with regard to multiracial identity (49% are white, 23% Black, 19% multiracial, 5% Latino, 1% Asian, 1% Native American, 3% undisclosed). Adolescent age was an average of 14.66 years. In terms of adolescent gender, our sample skewed male (37% female, 45% male, 7% nonbinary/gender nonconforming, 11% undisclosed), with 4% of the sample being transgender men and women (1% transgender female, 3% transgender male). Parent-reported adolescent sexual identity was also fairly diverse (40% heterosexual, 5% gay, 24% bisexual/pansexual, 6% lesbian, 7% asexual, 12% queer, 5% unknown or undisclosed by parent, and 1% identified as only attracted to female-presenting individuals).

When comparing participants gathered via Prolific versus those not gathered through Prolific, differences were present. Chi-squared tests demonstrated differences in terms of parent race *X*^2^ (5, *N* = 150) = 17.40, *p* = 0.004; parent gender *X*^2^ (7, *N* = 150) = 19.00, *p* = 0.003; and parent sexual identity *X*^2^ (7, *N* = 150) = 54.20, *p* < 0.001. Independent samples’ *t*-tests showed no differences in terms of parent age *t*(130) = 1.43, *p* = 0.08. Examining expected and observed values, the sample gathered from Prolific had a larger number of multiracial participants and fewer Black participants than would be expected. The Prolific sample also had fewer nonbinary and fewer transgender female participants and more cisgender male and female participants than would be expected. In terms of parent sexual identity, the Prolific sample had substantially more bisexual participants than would be expected and fewer gay and lesbian participants.

*T*-tests were used to examine differences between Prolific and snowball/community outreach participants in terms of the variables of interest. Differences were found in terms of GAD-7 *t*(131.8) = −2.14, *p* = 0.03; combined parent mental health *t*(133.1) = −2.1, *p* = 0.04; PSOC-LGBT *t*(144.2) = 1.99, *p* = 0.05; and PSI *t*(148) = 3.39, *p* < 0.001. This indicates that Prolific participants exhibit lower levels of anxiety, lower levels of combined mental health concerns, higher levels of sense of LGBTQ+ community, and higher levels of parenting stress. Due to these differences, we included recruitment method as a potential covariate.

### 2.2. Measures

#### 2.2.1. Parent Mental Health

To measure parental mental health, we used the Center for Epidemiological Studies Depression Scale (CES-D) and the Generalized Anxiety Disorder 7 item (GAD-7) to measure depression and anxiety severity, respectively. Both are widely used and standardized measures with good psychometric properties. Final depression and anxiety scores are also averaged to create a combined parent mental health score.

The CES-D [43] is a 20-question Likert scale measure with questions asking about the frequency of feelings such as “I was bothered by things that usually don’t bother me” with responses ranging from 1 to 4 (1 = rarely or none of the time (less than 1 day), 4 = most to all of the time (5–7 days)). Questions can involve both positive and negative feelings. A clinical cutoff is set at scores of 16 and above [43]. This measure was chosen in part due to its frequency of use within psychology [44] and in part due to the psychometric properties of the study, including its sensitivity and specificity [45], reliability [43,46], and validity [43]. The CES-D has been used in several studies with LGBTQ+ populations across a wide range of gender and sexual identities and locations across the U.S. [47,48,49].

The 7-item Generalized Anxiety Disorder Scale (GAD-7) [50] is a 7-question measure of general anxiety that operates on a 4-point Likert scale. Participants are asked about how often certain problems such as “Worrying too much about different things” have bothered them in the last 2 weeks (0 = not at all, 3 = nearly every day). A score of 0–4 indicates minimal anxiety, 5–9 mild anxiety, 10–14 moderate anxiety, and 15–21 severe anxiety (GAD-7) [50]. The GAD-7 also benefits from good reliability [50] and validity [50,51]. The GAD-7 has been used to examine the mental health of LGBTQ+ individuals across various identities [52] and has also been used outside of the West [53]. For our study, Cronbach’s alpha values illustrated high reliability across our mental health measures: CES-D α = 0.93, GAD-7 α = 0.92, and combined mental health scores α = 0.96.

#### 2.2.2. Parenting Stress

Parenting stress was measured via the Parenting Stress Index (PSI) Short Form [54], a 36-item Likert scale with 31 questions ranging from 1 to 5 (1 = strongly agree, 5 = strongly disagree) and 5 responses ranging from 1 to 5, with each utilizing question specific scales such as perceived quality of parenting (1 = not very good at being a parent, 5 = very good at being a parent). For this measure, we look at the total score of all PSI items. Higher scores indicate a higher level of parenting stress. This measure has been shown to have strong reliability and has been utilized previously across several different samples [55,56]. Across several different samples, we also see appropriate validity shown [55,57]. The PSI has been previously used in studies regarding LGBTQ+ parents spanning several family formation methods [25,26,58]. We found the PSI to exhibit high reliability in our study (α = 0.95).

#### 2.2.3. Child Adjustment

Child adjustment is a continuous variable measured via the Child Behavior Checklist (CBCL) for ages 6–18 [59]. The CBCL involves 113 Likert-scale questions with responses ranging from 1 to 3 (1 = not true, 3 = very true or often). Higher scores indicated worse child adjustment. Specifically, we analyze total behavioral problems, as well as examining the separate internalizing and externalizing scores. The CBCL normally features gender division in terms of which scores reach a normative versus clinical threshold. To make the measure more gender neutral and inclusive, we settled on scoring each participant as both male and female to determine a CBCL *T*-score and then choosing the larger of the 2 scores in line with previous gender inclusivity guidelines [60]. *T*-scores of 59 and lower indicate nonclinical symptoms. *T*-scores of 60–63 indicate borderline range, and scores of 64 and above indicate clinical ranges for internalizing behavior, externalizing behavior, and total child adjustment [59]. The CBCL is a quite ubiquitous measure chosen for its reliability, validity, and widespread use across a variety of samples [61]. Additionally, various age range versions of the CBCL have already been used effectively in LGBTQ+ parent research [24,62]. This measure illustrated high reliability in our study (α = 0.96).

#### 2.2.4. LGBTQ+ Community

Sense of LGBTQ+ community was measured via the Psychological Sense of LGBT Community Scale (PSOC-LGBT) [63]. Here, sense of community is defined as consisting of six components, which form the PSOC-LGBT: influence on others, influence from others, shared emotion, needs fulfillment, membership, and existence of community [63]. This scale is a 22-question Likert scale with responses ranging from 1 to 5 (1 = none, 5 = a great deal). The six subscales are each averaged, then the average of the subscales is determined to create a total score. This measure demonstrates appropriate reliability and validity [63] and has the distinction of being a measure that specifically assesses LGBTQ+ community belonging. The PSOC-LGBT has seen use in LGBTQ+ research both looking at LGBTQ+ community belonging within and outside of the U.S. [64,65]. This measure was found to be highly reliable within our study (α = 0.96).

### 2.3. Data Analysis Plan

Following data collection, we examined demographic data and associations among variables of interest. The planned analysis was to examine potential associations between these variables first via hierarchical regression and then follow up with a multiple-regression mode. Parent age, parent race, parent gender, parent sexual identity, child age, child race, if child is LGBTQ+, parent recruitment method (Prolific vs. snowball/community outreach), time of participation (i.e., we compared 2021 and 2022 versus 2023 and 2024 given the COVID-19 pandemic), and geographic location were all examined as potential covariates. Post hoc power analyses were run via G*Power Version 3.1.9.6 [66].

## 3. Results

### 3.1. Preliminary, Descriptive, and Post Hoc Power Analyses

Correlations were examined between variables of interest. Significant correlations were found between all variables with the exception of the PSOC-LGBT, which was not correlated with any variables. Significant correlations found were in the expected directions; parenting stress, child adjustment issues, and parent mental health were all positively correlated with one another (see Table 2).

Outliers in the model were identified as participants exhibiting significant issues in discrepancy, leverage, and/or influence utilizing standardized DfBetas, studentized deleted residuals, standardized DfFit, Cook’s D, and leverage values [67]. Participants who demonstrated issues in multiple areas were removed from the dataset, and analyses were rerun. This was repeated for a total of *n* = 2 individual participants when looking at the associations between PSI and CBCL, *n* = 1 PSI and mental health, and *n* = 2 when looking at the association between mental health and CBCL. For any model where multiple outliers were identified, the models were also run with all outliers removed. In all cases, significant results of our main variables of interest (PSI, CBCL, combined mental health, and PSOC) did not change. For this reason, we elected to include them in the final analysis. All values presented include these potential outliers.

Bivariate correlations and ANOVAs were run looking at associations between parent age, parent race, parent gender, parent sexual identity, child age, child race, if child is LGBTQ+, recruitment method, and geographic location with regard to our outcome variables. Additionally, since data were collected during the height of the COVID-19 pandemic, participants were separated into two groups (2021 and 2022 versus 2023 and 2024), and bivariate correlations were examined to see if lockdown procedures and heightened distress during the height of the pandemic impacted our outcome variables enough to be included in the model as a covariate. Variables that failed to demonstrate significant results with any outcome variables were removed from the final model. For the final model, parent age, parent race, parent sexual identity, parent recruitment method, and child race were kept as covariates. Parent and child race were collapsed down to a bivariate measure (white and person of color), and parent sexual identity was collapsed into five possible options which were dummy-coded for the final model with bisexual and pansexual/omnisexual combined to form a plurisexual category (different-gender attraction, same-gender attraction, plurisexual, asexual, queer). Participants were placed in the queer category if they chose queer when asked about sexual identity and chose no other options. With these covariates in place, the final models when run consisted of *n* = 132 participants.

Among all participants (*N* = 150), we found 40 (27%) participants gave CBCL scores for their adolescent children that were above the clinical range for total *T*-scores. Average CBCL *T*-scores were *M* = 55.64, which falls below the clinical and borderline thresholds [59]. Participants on average reported medium levels of feelings of LGBTQ+ sense of community (*M* = 3.36, maximum possible value = 5). Parent stress averaged *M* = 78.67 (maximum score of 180), parent depression averaged *M* = 12.77, falling below the clinical cutoff score of 16 [43], and parent anxiety averaged *M* = 5.89, which falls within the GAD-7 mild anxiety category [50]. Due to significant correlations between CES-D and GAD-7 scores (see Table 2), and these two variables being conceptually linked as mental health measures for the purposes of our study, we combined these measures into a combined mental health score. Average combined parent mental health scores were *M* = 10.35.

A one-way ANOVA was conducted to determine if sense of community varied across our four geographic regions. No significant difference was found in sense of LGBTQ+ community between geographic regions [*F*(3, 144) = 0.75, *p* = 0.524]. Additionally, following our analysis for potential covariates, we examined the relationship between parent sexual orientation and our outcome variables using analysis of variance. When looking at CBCL scores, there was a significant difference between groups, *F*(3, 144) = 3.60, *p* = 0.015. Sense of LGBTQ+ community was examined utilizing Welch and Brown–Forsythe tests due to lack of homogeneity of variance. Results indicated inconsistent findings (*p* = 0.064, and *p* = 0.024). Due to these inconsistencies, we did not probe this further. For all other outcome variables, no significant *p*-values were found. We used the Bonferroni correction to probe the significant ANOVA. Due to only having one different-gender-attracted individual in our sample, we removed that participant to allow for the correction (ANOVA values presented earlier reflect this omission). It is also worth noting that in our sample, asexual individuals constitute a small number of participants (*n* = 4). Final results of the Bonferroni correction found a significant difference between same-gender-attracted parents and plurisexual parents, with same-gender-attracted parents reporting better child adjustment (*p* = 0.018).

Post hoc power analyses were conducted using G*Power [66], models were examined, with parent and child race, parent sexual identity, parent age, and recruitment method included as covariates. When looking at the association between combined parent mental health on child adjustment we see a power of 0.99 (f^2^ = 0.22); similarly high power values are found when examining the association between parent stress and child adjustment issues (f^2^ = 0.52, power = 1) and when looking at the association between parent stress and combined parent mental health (f^2^ = 0.15, power = 0.98). When examining necessary power to determine a moderating effect, post hoc power analysis was performed looking at the PSOC-LGBT’s moderating effect on the relationship between combined parent mental health and child adjustment issues (f^2^ = 0.01, power = 0.18). Similarly low power values were found when examining the association between parent stress and child adjustment issues (f^2^ < 0.001, power = 0.05). Low power values were also found when looking at the association between parent stress and combined parent mental health (f^2^ < 0.001, power = 0.05). Together this demonstrates that at a sample size of *n* = 132, our study was well powered for direct effects but not to determine small-effect-size moderators. This is especially notable due to the difficult to reach the sample of our study.

### 3.2. Primary Analysis

Prior to attempting least squares multiple regression through an SPSS PROCESS macro [68], a series of hierarchical regressions were run to determine if adding sense of LGBTQ+ community to models had a significant impact. Parent age, parent race, child race, parent sexual identity (dummy-coded), and parent recruitment method were added to the models as covariates. Assumptions of homoscedasticity, normal distribution of residuals, and model fit were examined [69]. When looking at the association between PSI and CBCL, residuals were found to be skewed and kurtotic. When examining the association between PSI and mental health, residuals were found to be skewed. No other assumption violations were present. Due to the difficulties in interpreting transformed results, we elected to keep results untransformed. Issues in colinearity were identified but only between dummy-coded variables for parent sexual identity.

Results showed that controlling for our covariates, parent stress had a significant association with child adjustment issues (Table 3, *β* = 0.24, 95% CI [0.18, 0.30], *p* < 0.001, Δ*R*^2^ = 0.30) and the combined parent mental health score (Table 4, *β* = 0.12, 95% CI [0.06, 0.17], *p* < 0.001, Δ*R*^2^ = 0.11). Similarly, combined parent mental health was significantly associated with child adjustment issues (Table 5, *β* = 0.53, 95% CI [0.33, 0.74], *p* < 0.001, Δ*R*^2^ = 0.16). Results showed that higher levels of parenting stress were associated with greater child adjustment issues and greater combined parent mental health concerns and that greater combined parent mental health issues were associated with greater child adjustment issues. However, in each case, adding sense of LGBTQ+ community to the model failed to yield a significant Δ*R*^2^. Similarly, adding the cross-product of PSOC-LGBT and the IV failed to yield a significant Δ*R*^2^. For this reason, the models including the PSOC-LGBT as a moderator were not used. In each case, the models were run with CES-D and GAD-7 each in place of combined mental health. For each applicable model, the main effects of PSI on mental health and mental health on CBCL were still significant.

Parent age, parent race (white or person of color), child race (white or person of color), parent sexual identity (different-gender attraction, same-gender attraction, plurisexual, asexual, or queer), and recruitment method (Prolific or snowball sampling/community outreach) were examined as potential covariates. When examining the relationship between parenting stress and child adjustment issues, plurisexual parents reported worse CBCL scores than same-gender-attracted parents (*β* = 4.47, 95% CI [0.65, 8.30], *p* = 0.022), as did asexual parents (*β* = 11.18, 95% CI [1.28, 21.09], *p* = 0.027). Additionally, queer parents reported better scores than asexual parents (*β* = −11.74, 95% CI [−22.91, −0.57], *p* = 0.040). Parents recruited through Prolific reported worse CBCL scores than parents recruited through snowball sampling/community outreach (*β* = 4.22, 95% CI [0.42, 8.02], *p* = 0.030). When examining the relationship between parenting stress and combined parent mental health, age acted as a covariate, with older parent age being associated with better mental health (*β* = −0.23, 95% CI [−0.38, −0.07], *p* = 0.005). When examining the relationship of combined parent mental health and child adjustment issues, plurisexual parents (*β* = 5.06, 95% CI [0.81, 9.32] *p* = 0.020) and asexual parents (*β* = 12.46, 95% CI [1.41, 23.51] *p* = 0.027) reported worse mental health than same-gender attracted parents.

With the prior literature suggesting a mediating role of parent depression (measured partially via CES-D) in general samples when examining the relationship between parent stress and child adjustment [70], we sought to examine if this model would be applicable when looking at parenting stress specifically and within a LGBTQ+ parent context. Post hoc analyses were run examining parent mental health as a potential mediator of the relationship between parent stress and child adjustment. Parent age, parent sexual orientation, parent race, parent recruitment method, and child race were included as covariates. An SPSS PROCESS macro [68] was utilized. Results indicated partial mediation. A significant direct effect was found from parenting stress to child adjustment issues (*β* = 0.20, 95% bootstrapped CI [0.14, 0.26], *p* < 0.001). A significant indirect effect was also found with a significant a path from PSI to combined mental health score (*β* = 0.12, 95% bootstrapped CI [0.06, 0.17], *p* = 0.001) and a significant b path from combined parent mental health to CBCL score (*β* = 0.31, 95% bootstrapped CI [0.13, 0.50], *p* = 0.001). Results indicate not only that higher levels of parenting stress are associated with worse child adjustment but also that parent mental health partially mediated this relationship such that increased parenting stress is associated with worse parent mental health, which is then associated with worse child adjustment.

Our first hypothesis was supported, with greater parenting stress associated with worse child adjustment and worse parent mental health. Worse child adjustment was also associated with worse parent mental health. However, hypothesis 2 was not supported, as sense of LGBTQ+ community did not statistically impact the models. Post hoc analysis did provide unique information with regard to how parenting stress interacts with child adjustment, with parent mental health mediating the association. This finding illustrates the complexity of the relationship, emphasizing the need to examine several mental health variables when examining the family system and further emphasizing the need to value LGBTQ+ parent mental health.

## 4. Discussion

In this study, we hypothesized that when looking at LGBTQ+ parents of adolescent children, we would see a positive association between the parent’s negative mental health, parenting stress, and issues with child adjustment. We also hypothesized that the parent’s sense of LGBTQ+ community would moderate these associations, acting as a protective factor. Partial support for these hypotheses was found. Results indicated that parent stress, parent mental health, and child adjustment all interact in a way consistent with Ecological Systems Theory [12], which emphasizes the interplay between parent, child, and the environment. That is, parent stress was associated with worse parent mental health, and both parent stress and worse parent mental health were associated with worse child adjustment. These results do not identify causation. However, in line with Ecological Systems Theory [12], we would expect these relationships to be reciprocal in nature. These results further support similarities between LGBTQ+- and cis-het-parented families. Prior cis-het or general sample research indicates links between parent stress and parent mental health [71,72,73], parent stress and child adjustment [19,20], and child adjustment and parent mental health [35,74]. Our findings mirror these results. Post hoc analysis demonstrated a unique relationship between these three variables, with parent mental health mediating the relationship between PSI and CBCL. Prior research has found that maternal parenting stress acts as a mediator when looking at the relationship between anxiety and child adjustment [75]. However, in this study, we conceptualize mental health concerns as being the mediating factor in our model in line with prior findings utilizing the CES-D [70]. We conceptualize parenting stress as a factor influencing parent mental health rather than the other way around. However, it is likely the relationship is actually reciprocal. Future experimental work could expand upon this to determine what variable is appropriate as the independent variable versus being the mediator. Our post hoc findings emphasize the interconnectivity of these constructs and the need for researchers to examine the family system across several measures to illustrate a more complete picture of family well-being. These findings are also notable as the literature is presently lacking with regard to LGBTQ+ parent research that specifically looks at parents with adolescent children, as well as a general lack of U.S. LGBTQ+ research outside of coastal regions. This research is also notable for having a diverse sample of participants with regard to race, gender, and sexual identity. Our study had a substantial number of participants who identified as a person of color (36%), transgender, nonbinary or otherwise outside of the gender binary (15%), and plurisexual (55%).

Our hypothesis that the parent’s sense of LGBTQ+ community would moderate the associations between PSI, CBCL, and combined parent mental health, was not supported. Results failed to illustrate a moderating effect of sense of LGBTQ+ community, and lack of correlations between sense of LGBTQ+ community and all other variables suggests a general lack of relation. This contrasts Ecological Systems Theory’s [12] emphasis on the role of the mesosystem. This theory suggests that community could have a strong impact on the family system, but our results do not support such an impact, at least among the variables we considered. This also contrasts with prior research findings that connection to the LGBTQ+ community plays an important role when examining LGBTQ+ mental health [13,14,18]. While lack of significance cannot be truly interpreted, the lack of findings could be due to other factors such as governmental support being more influential, or parent’s connection to the LGBTQ+ community being less salient than parent identity and their connection to their child, at least as connected to parent mental health, parenting stress, and adolescent adjustment. It is also possible that participants may have a more ambivalent feeling towards LGBTQ+ community, with negative and positive aspects balancing each other out, leading to a less clear measure of sense of community. Notably this study focuses on parents of adolescent children. Perhaps this led to the lack of significant moderation among variables of interest. It may be that sense of LGBTQ+ community is more important when children are young and parents are more likely to directly involve them with the LGBTQ+ community. With adolescence being a key time for child autonomy [76], perhaps the parent’s sense of LGBTQ+ community is less relevant. It is also possible that the COVID-19 pandemic impacted LGBTQ+ community connections, as our data collection started in 2021. However, we did not find significant associations between participating in this study during the height of the pandemic (2021–2022 versus 2023–2024) and our outcome variables.

Results also demonstrated noteworthy findings with regard to individual identity differences. Results indicated that asexual and plurisexual parents reported worse child outcomes, specifically when compared to same-gender-attracted parents and, in the case of asexual parents, parents who identified solely as queer. These findings relate to prior research from both within and outside of the U.S. that demonstrated bisexual individuals face worse mental health outcomes than same-gender-attracted individuals [18,77,78], as well as research finding greater stigma reported by asexual participants when compared to non-asexual LGBTQ+ participants [79]. Our study builds upon this literature by identifying these dynamics in an entirely parent sample, and these findings can translate to parent-reported CBCL score. However, it is notable that these covariate effects were not found when examining parent combined mental health, which does not align with past research. This may indicate that prejudice towards plurisexual and asexual parents is uniquely felt by their children even when parents are not impacted.

For plurisexual individuals in our sample, we had participants across a wide range of relationship types (same-gender, different-gender, polyamorous, etc.) and relationship statuses. It is possible that plurisexual individuals with partners not accepting of their LGBTQ+ identity, and parents who are currently concealing their LGBTQ+ identity from their children, may explain why we see negative outcomes with plurisexual individuals. Asexual and plurisexual identities are unique when compared to same-gender attraction in how concealable they may be to the participants’ children. While not able to be fully explored in this study, it is possible that identity concealment or identity-related conflict within the relationship may account for negative outcomes found with regard to sexual identity. These unique findings encourage further exploration into how stigma and prejudice may impact the family system, particularly when examining the experiences of individuals with different LGBTQ+ identities.

Our findings of sense of LGBTQ+ community being present within Southern and Midwest states, and not significantly varying across geographic regions in the U.S., are also notable (though in the latter case, nonsignificant results cannot truly be interpreted). With self-determination theory [80] emphasizing the importance of relatedness, sense of community is of key importance when looking at LGBTQ+ families. While LGBTQ+ families in the South may live in states with harmful laws [81], this study suggests that despite this, LGBTQ+ community is still perceived within the South.

This study also offers some interesting findings regarding data collection methods. Differences in sexual identities across groups may indicate that snowball and community-based sampling has less ability to reach bisexual individuals. With several of the Prolific recruited bisexual individuals being in different-gender relationships (often marriage), it seems that a unique benefit of Prolific recruitment is being able to reach LGBTQ+ individuals in different-gender relationships who may otherwise not be active in LGBTQ+ spaces enough that community outreach would reach them. Recruitment method was also found to act as a covariate in one of our three models. These findings identify the potential skew that can be present when recruiting from a single source.

### 4.1. Research Limitations

Our study was purely focused on cross-sectional survey data. As such, we are unable to determine causation from our results. Additionally, our study focused on a specific subsample LGBTQ+ individuals. As such, we were limited in our recruitment, leading to a study low in power when looking for moderating effects. Lastly, the PSOC-LGBT [54] measuring sense of community rather than community participation could also be an issue. A measure that looks more into community activity and involvement may be a more appropriate measure and one that may act as a moderator.

In terms of our findings related to LGBTQ+ identity, it is notable that the covariate effects were not found when examining parent combined mental health, which does not align with past research [18,77,78,79]. This may have occurred because the mental health outcomes examined in this study do not illustrate the full picture of how parent mental health is impacted by prejudice. It is also worth noting that there were few asexual individuals (*n* = 4), and we did not find asexual participants to significantly differ in our outcome variables from other sexual identity groups when running ANOVAs. As such the findings with regard to asexual individuals should be viewed with a level of scrutiny.

### 4.2. Future Directions

With our lack of significant moderation, perhaps future research should utilize different measures of community. Additionally, more in-depth models may be worth examining, such as a moderation model where community engagement could then itself be potentially moderated (three-way interaction) by the parent’s belief that the LGBTQ+ community is accepting and inclusive of parents. It may also be prudent for future research to examine how the PSOC-LGBT relates to other outcomes in LGBTQ+ parents such as readiness to engage in pro-LGBTQ+ activism and children’s sense of community. The latter of these constructs ties into parent socialization, which may also play a role in child adjustment.

This study sought to focus on Midwest and Southern LGBTQ+ individuals as they are often underserved in research [9]. We believe that further research is needed within these regions, and future research could build upon this paper by examining how LGBTQ+ community is formed in states and regions where LGBTQ+ rights are under siege. Our findings also suggest that differences in parent-reported CBCL scores may differ based on recruitment method, illustrating the importance of collecting data through various methods when possible.

Our findings also demonstrate that parent sexual identity can impact the family system. We believe that this is due to the unique stigma members of these groups may face. Future research should examine these differences, perhaps utilizing qualitative data to gather a more robust picture of the life experiences of LGBTQ+ parents who hold identities that may face greater stigma.

### 4.3. Implications for Practice, Policy, and Law

This study’s findings emphasize the importance of attending to the mental well-being of LGBTQ+ parents and their children. With regard to clinical practice, these findings illustrate the need to attend to the larger family system when tackling parent or adolescent mental health concerns. This study also highlights the prevalence of sense of LGBTQ+ community even within regions that may have less supportive laws in place. At a time when the rights of LGBTQ+ people are under attack [81,82] and LGBTQ+ community-centric pride events have become controversial, if not dangerous [83,84], it is important for research to highlight that not only do LGBTQ+ parents raise healthy, well-adjusted children but that LGBTQ+ community can exist outside of the more frequently researched areas of the United States.

## 5. Conclusions

This study examined the inner workings of the family system, focusing on a unique sample within areas of the U.S. underserved in past research [9]. Partial support for our hypotheses was found: higher parenting stress was associated with worse parent mental health, worse parent mental health was associated with worse child adjustment, and higher parenting stress was associated with worse child adjustment. Additionally, a post hoc analysis demonstrated that the association between parenting stress and child adjustment was mediated by parent mental health. Greater parenting stress was associated with worse child adjustment. Partial mediation was also present with parenting stress associated with greater mental health issues, which was then associated with worse child adjustment. These findings align with prior research on cis-het-parented families and general samples [19,20,25,61,62,63,64,65] but now do so in a 100% LGBTQ+ sample, and within a sample that is diverse in racial, sexual, and gender identity. Within LGBTQ+ parent research, our study is also notable for focusing on parents of adolescent children specifically, a population that is less often examined within the larger body of LGBTQ+ parent research. Overall, this study examines members of the LGBTQ+ community who may not have had their well-being examined in other studies.

Parent sexual identity was found to be a covariate in all models with the CBCL present as a dependent variable, with asexual and plurisexual parents reporting worse child adjustment outcomes compared to same-gender-attracted and asexual parents reporting lower CBCL scores than queer parents in one model. These findings emphasize the unique experiences of various LGBTQ+ identities and highlight a need for future research examining these different outcomes in greater detail. Age was also shown to play a role in parent’s mental health, with older age being associated with better mental health in line with prior general sample research [32].

As LGBTQ+ rights continue to be under fire [81,82], this research emphasizes that to protect the well-being of LGBTQ+ parents and their children, we need to take note of the entire family system. The present findings illustrated the importance of LGBTQ+ parent mental health when discussing child well-being. This is in opposition to arguments that LGBTQ+ parents are inherently worse parents or that it is irresponsible for LGBTQ+ individuals to become parents [2]. Instead, this research illustrates that protecting the mental health of LGBTQ+ families is key. This may be especially important in adolescent children as adolescent mental health is a key area of concern within the U.S. [85]. Our findings suggest that LGBTQ+ parents can have children with good child adjustment, but parent mental health and parenting stress are important factors to observe and address. Together the results of this study emphasize the importance of LGBTQ+ parent mental health, illustrate the similarities between LGBTQ+-parented families and cis-het-parented families, and shine a light on both the unique way the relationship between parent stress and child adjustment is mediated by parent mental health and the unique impact age and sexual identity-related stigma can have on LGBTQ+-parented families.

## Figures and Tables

**Table 1:** Parent demographics.

Characteristic	Total	Percent
**Gender**		
Cisgender woman	83	55%
Cisgender man	43	29%
Transgender woman	7	5%
Transgender man	2	2%
Genderqueer/nonbinary	11	7%
Gender nonconforming	1	1%
Pangender/genderfluid	2	2%
Undisclosed	1	1%
**Race/Ethnicity**		
White	96	64%
Black	35	23%
Latinx	3	2%
Asian	1	1%
Native American	1	1%
Multiracial	14	9%
**Sexual Identity**		
Gay	23	15%
Lesbian	29	19%
Bisexual	66	44%
Queer	10	7%
Asexual	4	3%
Pansexual/omnisexual	16	11%
Different-gender attracted	1	1%
Undisclosed	1	1%
**Geography**		
South	65	43%
Midwest	39	26%
Northeast	24	16%
West	22	15%

**Table 2:** Correlations.

PSI	-					
CBCL	0.50 ***	-				
CES-D	0.33 ***	0.44 ***	-			
GAD-7	0.28 ***	0.42 ***	0.81 ***	-		
Combined Mental Health	0.32 ***	0.45 ***	0.98 ***	0.91 ***	-	
PSOC-LGBT	−0.06	−0.04	−0.13	−0.07	−0.11	-

*** indicates *p* < 0.001.

**Table 3:** Regression model PSI on CBCL.

	Model 1	Model 2	Model 3	Model 4
Parent different-gender attracted	-	-	-	-
Parent same-gender attracted	4.01	-	-	-
Parent plurisexual	8.49	4.47 *	-	-
Parent asexual	15.20	11.18 *	6.71	-
Parent queer	3.45	−0.56	−5.03	−11.74 *
Parent is white	1.28			
Child is white	−1.91			
Parent age	−0.06			
Recruited through prolific	4.22 *			
PSI	0.24 ***			
*R* ^2^	0.42			
∆*R*^2^ when adding PSI	0.30 ***			

DV = CBCL. Model 1: reference group parent different-gender attracted. Model 2: reference group parent same-gender attracted. Model 3: reference parent plurisexual. Model 4: Reference group parent asexual. Parent queer indicates parent used queer as their only sexual identity identification. * indicates *p* < 0.05. *** indicates *p* < 0.001.

**Table 4:** Regression model PSI on mental health.

	Model 1	Model 2	Model 3	Model 4
Parent different-gender attracted	-	-	-	-
Parent same-gender attracted	1.12	-	-	-
Parent plurisexual	2.42	1.29	-	-
Parent asexual	−0.45	−1.58	−2.87	-
Parent queer	1.72	0.60	−0.70	2.18
Parent is white	2.75			
Child is white	−0.56			
Parent age	−0.23 **			
Recruited through prolific	3.38			
PSI	0.12 ***			
*R* ^2^	0.26			
∆*R*^2^ when adding PSI	0.11 ***			

DV = Combined parent mental health. Model 1: reference group parent different-gender attracted. Model 2: reference group parent same-gender attracted. Model 3: reference parent plurisexual. Model 4: Reference group parent asexual. Parent queer indicates parent used queer as their only sexual identity identification. ** indicates *p* < 0.01. *** indicates *p* < 0.001.

**Table 5:** Regression model mental health on CBCL.

	Model 1	Model 2	Model 3	Model 4
Parent different-gender attracted	-	-	-	-
Parent same-gender attracted	1.96	-	-	-
Parent plurisexual	7.03	5.06 *	-	-
Parent asexual	14.42	12.46 *	7.39	-
Parent queer	2.55	0.59	−4.47	−11.87
Parent is white	−0.06			
Child is white	0.05			
Parent age	0.001			
Recruited through prolific	−0.91			
Mental health	0.53 ***			
*R* ^2^	0.28			
∆*R*^2^ when adding mental health	0.16 ***			

DV = CBCL. Model 1: reference group parent different-gender attracted. Model 2: reference group parent same-gender attracted. Model 3: reference parent plurisexual. Model 4: reference group parent asexual. Parent queer indicates parent used queer as their only sexual identity identification. * indicates *p* < 0.05. *** indicates *p* < 0.001.

## Data Availability

The datasets presented in this article are not readily available because data is still being utilized for ongoing studies.

## References

[1] American Psychological Association (2020). Resolution on Sexual Orientation, Gender Identity (Sogi), Parents, and Their Children. https://www.apa.org/pi/lgbt/resources/policy/sexual-orientation.

[2] Clarke V. (2001). What about the children? Arguments against lesbian and gay parenting. Women’s Stud. Int. Forum.

[3] Family Equality Council Family Equality’s Work in the Courts. Family Equality.

[4] Levitt H.M., Schuyler S.W., Chickerella R., Elber A., White L., Troeger R.L., Karter J.M., Preston J.M., Collins K.M. (2020). How discrimination in adoptive, foster, and medical systems harms LGBTQ+ families: Research on the experiences of prospective parents. J. Gay Lesbian Soc. Serv..

[5] Movement Advancement Project (n.d.) Equality Maps Snapshot: LGBTQ Equality by State. Movement Advancement Project. https://www.mapresearch.org/equality-maps/.

[6] Movement Advancement Project (2019). Where We Call Home: LGBT People in Rural America. Movement Advancement Project. www.lgbtmap.org/rural-lgbt.

[7] National Association for the Advancement of Colored People NAACP Issues Travel Advisory in Florida. 20 May 2023. https://naacp.org/articles/naacp-issues-travel-advisory-florida.

[8] Vaughan M.D., Rodriguez E.M. (2014). LGBT strengths: Incorporating positive psychology into theory, research, training, and practice. Psychol. Sex. Orientat. Gend. Divers..

[9] Stone A.L. (2018). The Geography of Research on LGBTQ Life: Why sociologists should study the South, rural queers, and ordinary cities. Sociol. Compass.

[10] Upreti R. (2017). Identity Construction: An Important Issue Among Adolescents. IOSR J. Humanit. Soc. Sci..

[11] Goldberg A.E., Kinkler L.A., Richardson H.B., Downing J.B. (2012). On the border: Young adults with LGBQ parents navigate LGBTQ communities. J. Couns. Psychol..

[12] Bronfenbrenner U. (1977). Toward an experimental ecology of human development. Am. Psychol..

[13] Kaniuka A., Pugh K.C., Jordan M., Brooks B., Dodd J., Mann A.K., Williams S.L., Hirsch J.K. (2019). Stigma and suicide risk among the LGBTQ population: Are anxiety and depression to blame and can connectedness to the LGBTQ community help?. J. Gay Lesbian Ment. Health.

[14] Frost D.M., Meyer I.H., Lin A., Wilson B.D., Lightfoot M., Russell S.T., Hammack P.L. (2022). Social change and the health of sexual minority individuals: Do the effects of minority stress and community connectedness vary by age cohort?. Arch. Sex. Behav..

[15] Austin A., Craig S.L., Navega N., McInroy L.B. (2020). It’s my safe space: The life-saving role of the internet in the lives of transgender and gender diverse youth. Int. J. Transgender Health.

[16] DeHaan S., Kuper L.E., Magee J.C., Bigelow L., Mustanski B.S. (2013). The Interplay between Online and Offline Explorations of Identity, Relationships, and Sex: A Mixed-Methods Study with LGBT Youth. J. Sex Res..

[17] Power J., Brown R., Schofield M.J., Pitts M., McNair R., Perlesz A., Bickerdike A. (2014). Social connectedness among lesbian, gay, bisexual, and transgender parents living in metropolitan and regional and rural areas of Australia and New Zealand. J. Community Psychol..

[18] Chan R.C.H., Operario D., Mak W.W.S. (2020). Bisexual individuals are at greater risk of poor mental health than lesbians and gay men: The mediating role of sexual identity stress at multiple levels. J. Affect. Disord..

[19] The Trevor Project (2021). LGBTQ Youth in the South. https://www.thetrevorproject.org/research-briefs/lgbtq-youth-in-the-south-dec-2021/.

[20] Harless C., Nanney M., Johnson A.H., Polaski A., Beach-Ferrara J. (2019). The Report of the 2019 Southern LGBTQ Health Survey.

[21] Pew Research Center (2015). Religious Landscape Study. Pew Research Center’s Religion & Public Life Project.

[22] Stone A.L. (2016). The Impact of Anti-Gay Politics on the LGBTQ Movement. Sociol. Compass.

[23] Movement Advancement Project (2020). LGBTQ Policy Spotlight: Mapping LGBTQ Equality in the U.S. South. Movement Advance-Ment Project. www.lgbtmap.org/regional-south-tally.

[24] Goldberg A.E., Smith J.Z. (2013). Predictors of psychological adjustment in early placed adopted children with lesbian, gay, and heterosexual parents. J. Fam. Psychol..

[25] Golombok S., Mellish L., Jennings S., Casey P., Tasker F., Lamb M.E. (2014). Adoptive Gay Father Families: Parent–Child Relationships and Children’s Psychological Adjustment. Child Dev..

[26] Goldberg A.E., Smith J.Z. (2014). Predictors of Parenting Stress in Lesbian, Gay, and Heterosexual Adoptive Parents during Early Parenthood. J. Fam. Psychol. JFP J. Div. Fam. Psychol. Am. Psychol. Assoc..

[27] Goldberg A.E., Smith J.Z., McCormick N.M., Overstreet N.M. (2019). Health behaviors and outcomes of parents in same-sex couples: An exploratory study. Psychol. Sex. Orientat. Gend. Divers..

[28] Östberg M., Hagekull B. (2013). Parenting stress and external stressors as predictors of maternal ratings of child adjustment. Scand. J. Psychol..

[29] Rodriguez C.M. (2011). Association Between Independent Reports of Maternal Parenting Stress and Children’s Internalizing Symptomatology. J. Child Fam. Stud..

[30] Ahmadzadeh Y.I., Schoeler T., Han M., Pingault J.-B., Creswell C., McAdams T.A. (2021). Systematic Review and Meta-analysis of Genetically Informed Research: Associations Between Parent Anxiety and Offspring Internalizing Problems. J. Am. Acad. Child Adolesc. Psychiatry.

[31] Cummings E.M., Davies P.T. (1994). Maternal depression and child development. J. Child Psychol. Psychiatry.

[32] Downey G., Coyne J.C. (1990). Children of depressed parents: An integrative review. Psychol. Bull..

[33] Hammen C., Brennan P.A. (2003). Severity, chronicity, and timing of maternal depression and risk for adolescent offspring diagnoses in a community sample. Arch. Gen. Psychiatry.

[34] Lawrence P.J., Murayama K., Creswell C. (2019). Systematic Review and Meta-Analysis: Anxiety and Depressive Disorders in Offspring of Parents with Anxiety Disorders. J. Am. Acad. Child Adolesc. Psychiatry.

[35] Bos H.M.W., Gartrell N.K., Peyser H., Van Balen F. (2008). The USA National Longitudinal Lesbian Family Study (NLLFS): Homophobia, Psychological Adjustment, and Protective Factors. J. Lesbian Stud..

[36] Brooks V.R. (1981). Minority Stress and Lesbian Women.

[37] Meyer I.H. (2003). Prejudice, social stress, and mental health in lesbian, gay, and bisexual populations: Conceptual issues and research evidence. Psychol. Bull..

[38] Parkerson H.A., Thibodeau M.A., Brandt C.P., Zvolensky M.J., Asmundson G.J. (2015). Cultural-based biases of the GAD-7. J. Anxiety Disord..

[39] Nomaguchi K., House A.N. (2013). Racial-ethnic disparities in maternal parenting stress: The role of structural disadvantages and parenting values. J. Health Soc. Behav..

[40] Tabler J., Geist C., Schmitz R.M., Nagata J.M. (2019). Does it get better? Change in depressive symptoms from late-adolescence to early-adulthood, disordered eating behaviors, and sexual identity. J. Gay Lesbian Ment. Health.

[41] Sequeira S.L., Morrow K.E., Silk J.S., Kolko D.J., Pilkonis P.A., Lindhiem O. (2021). National norms and correlates of the PHQ-8 and GAD-7 in parents of school-age children. J. Child Fam. Stud..

[42] Henderson A.S., Jorm A.F., Korten A.E., Jacomb P., Christensen H., Rodgers B. (1998). Symptoms of depression and anxiety during adult life: Evidence for a decline in prevalence with age. Psychol. Med..

[43] Radloff L.S. (1977). The CES-D Scale: A Self-Report Depression Scale for Research in the General Population. Appl. Psychol. Meas..

[44] Santor D.A., Gregus M., Welch A. (2006). Focus Article: Eight Decades of Measurement in Depression. Meas. Interdiscip. Res. Perspect..

[45] Shean G., Baldwin G. (2008). Sensitivity and Specificity of Depression Questionnaires in a College-Age Sample. J. Genet. Psychol..

[46] Devins G., Orme C., Costello C., Binik Y., Frizzell B., Stam H., Pullin W. (1988). Measuring depressive symptoms in illness populations: Psychometric properties of the Center for Epidemiologic Studies Depression (CES-D) Scale. Psychol. Health.

[47] Hightow-Weidman L.B., Phillips G., Jones K.C., Outlaw A.Y., Fields S.D., Smith for TY of CS IS G., Justin C. (2011). Racial and Sexual Identity-Related Maltreatment Among Minority YMSM: Prevalence, Perceptions, and the Association with Emotional Distress. AIDS Patient Care and STDs.

[48] McCarthy M.A., Fisher C.M., Irwin J.A., Coleman J.D., Pelster A.D.K. (2014). Using the Minority Stress Model to Understand Depression in Lesbian, Gay, Bisexual, and Transgender Individuals in Nebraska. J. Gay Lesbian Ment. Health.

[49] Russell S.T., Ryan C., Toomey R.B., Diaz R.M., Sanchez J. (2011). Lesbian, Gay, Bisexual, and Transgender Adolescent School Victimization: Implications for Young Adult Health and Adjustment. J. Sch. Health.

[50] Spitzer R.L., Kroenke K., Williams J.B.W., Löwe B. (2006). A Brief Measure for Assessing Generalized Anxiety Disorder: The GAD-7. Arch. Intern. Med..

[51] Löwe B., Decker O., Müller S., Brähler E., Schellberg D., Herzog W., Herzberg P.Y. (2008). Validation and Standardization of the Generalized Anxiety Disorder Screener (GAD-7) in the General Population. Med. Care.

[52] Borgogna N.C., McDermott R.C., Aita S.L., Kridel M.M. (2019). Anxiety and depression across gender and sexual minorities: Implications for transgender, gender nonconforming, pansexual, demisexual, asexual, queer, and questioning individuals. Psychol. Sex. Orientat. Gend. Divers..

[53] Wang Y., Feng Y., Han M., Duan Z., Wilson A., Fish J., Sun S., Chen R. (2021). Methods of attempted suicide and risk factors in LGBTQ+ youth. Child Abus. Negl..

[54] Abidin R.R. (1995). Parenting Stress Index.

[55] Aracena M., Gómez Muzzio E., Undurraga C., Leiva L., Marinkovic Chavez K., Molina Y. (2016). Validity and Reliability of the Parenting Stress Index Short Form (PSI-SF) Applied to a Chilean Sample. J. Child Fam. Stud..

[56] Ghosh Ippen C., Kuendig C., Mayorga L. (2014). Parenting Stress Index Short Form. National Child Traumatic Stress Network Measure Review Database. https://www.nctsn.org/measures/parenting-stress-index-short-form.

[57] Lee S.J., Gopalan G., Harrington D. (2016). Validation of the Parenting Stress Index–Short Form with Minority Caregivers. Res. Soc. Work Pract..

[58] Tornello S.L., Patterson C.J. (2012). Gay Fathers in Mixed-Orientation Relationships: Experiences of Those Who Stay in Their Marriages and of Those Who Leave. J. GLBT Fam. Stud..

[59] Achenbach T.M., Dumenci L., Rescorla L.A. (2001). Ratings of relations between DSM-IV diagnostic categories and items of the CBCL/6-18, TRF, and YSR.

[60] Achenbach T.M., Ivanova M.Y. (2022). Guide to Gender-Inclusive Assessment Using the ASEBA.

[61] Thorvaldsen M., Taylor N., Igelmen R., Kulkarni M., Ghosh Ippen C. (2013). Child Behavior Checklist for Ages 6–18. National Child Traumatic Stress Network Measure Review Database. https://www.nctsn.org/measures/child-behavior-checklist-ages-6-18.

[62] Gartrell N., Rodas C., Deck A., Peyser H., Banks A. (2005). The national lesbian family study: 4. Interviews with the 10-year-old children. Am. J. Orthopsychiatry.

[63] Lin Y., Israel T. (2012). Development and validation of a Psychological Sense of LGBT Community Scale. J. Community Psychol..

[64] Cogger A., Conover K.J., Israel T. (2012). Factors Influencing Alcohol Use Among Sexual Minority Women in a Non-Urban Community: A Mixed Methods Study. J. LGBT Issues Couns..

[65] Zervoulis K., Smith D.S., Reed R., Dinos S. (2020). Use of ‘gay dating apps’ and its relationship with individual well-being and sense of community in men who have sex with men. Psychol. Sex..

[66] Faul F., Erdfelder E., Buchner A., Lang A.-G. (2009). Statistical power analyses using G*Power 3.1: Tests for correlation and regression analyses. Behav. Res. Methods.

[67] Aguinis H., Gottfredson R.K., Joo H. (2013). Best-practice recommendations for defining, identifying, and handling outliers. Organ. Res. Methods.

[68] Hayes A.F. (2017). Introduction to Mediation, Moderation, and Conditional Process Analysis: A Regression-Based Approach.

[69] Osborne J.W., Waters E. (2019). Four assumptions of multiple regression that researchers should always test. Pract. Assess. Res. Eval..

[70] Conger R.D., Patterson G.R., Ge X. (1995). It takes two to replicate: A mediational model for the impact of parents’ stress on adolescent adjustment. Child Dev..

[71] Cornish A.M., McMahon C.A., Ungerer J.A., Barnett B., Kowalenko N., Tennant C. (2006). Maternal depression and the experience of parenting in the second postnatal year. J. Reprod. Infant Psychol..

[72] Farmer A.Y., Lee S.K. (2011). The Effects of Parenting Stress, Perceived Mastery, and Maternal Depression on Parent–Child Interaction. J. Soc. Serv. Res..

[73] Respler-Herman M., Mowder B.A., Yasik A.E., Shamah R. (2012). Parenting beliefs, parental stress, and social support relationships. J. Child Fam. Stud..

[74] Woodruff-Borden J., Morrow C., Bourland S., Cambron S. (2002). The behavior of anxious parents: Examining mechanisms of transmission of anxiety from parent to child. J. Clin. Child Adolesc. Psychol..

[75] Tsotsi S., Broekman B.F., Sim L.W., Shek L.P., Tan K.H., Chong Y.S., Rifkin-Graboi A. (2019). Maternal anxiety, parenting stress, and preschoolers’ behavior problems: The role of child self-regulation. J. Dev. Behav. Pediatr..

[76] Zimmer-Gembeck M.J., Collins W.A. (2006). Autonomy development during adolescence. Blackwell Handbook of Adolescence.

[77] Fredriksen-Goldsen K.I., Kim H.J., Barkan S.E., Balsam K.F., Mincer S.L. (2010). Disparities in health-related quality of life: A comparison of lesbians and bisexual women. Am. J. Public Health.

[78] Kerr D.L., Santurri L., Peters P. (2013). A comparison of lesbian, bisexual, and heterosexual college undergraduate women on selected mental health issues. J. Am. Coll. Health.

[79] Rothblum E.D., Krueger E.A., Kittle K.R., Meyer I.H. (2020). Asexual and non-asexual respondents from a US population-based study of sexual minorities. Arch. Sex. Behav..

[80] Ryan R.M., Deci E.L. (2000). Self-determination theory and the facilitation of intrinsic motivation, social development, and well-being. Am. Psychol..

[81] ACLU (2023). Doe v. Abbott American Civil Liberties Union.

[82] Hassan A. (2023). States Passed a Record Number of Transgender Laws. Here’s What They Say. The New York Times.

[83] Soplesa B. (2023). Kansas Man Arrested and Charged with Threatening to Bomb Nashville Pride Event. NBC News.

[84] Sullivan B. (2022). A Far-Right Plan to Riot near an Idaho LGBTQ Event Heightens Safety Concerns at Pride. NPR.

[85] Center for Disease Control and Prevention (2021). Youth Risk Behavior Survey Data Summary and Trends Report 2011–2021. https://www.cdc.gov/healthyyouth/data/yrbs/yrbs_data_summary_and_trends.htm.

